# Curative Resection of Locally Advanced Colon Cancer that Invading the Common Iliac Artery by Creating an Extra-Anatomical Arterial Bypass: A Case Report

**DOI:** 10.70352/scrj.cr.25-0125

**Published:** 2025-06-21

**Authors:** Tatsuki Noguchi, Yosuke Fukunaga, Toshio Takayama, Takashi Sakamoto, Shimpei Matsui, Toshiki Mukai, Tomohiro Yamaguchi, Manabu Takamatsu, Takashi Akiyoshi

**Affiliations:** 1 Department of Colorectal Surgery, Gastroenterological Center, Cancer Institute Hospital, Japanese Foundation for Cancer Research, Tokyo, Japan; 2 Rectal Cancer Multidisciplinary Treatment Center, Cancer Institute Hospital, Japanese Foundation for Cancer Research, Tokyo, Japan; 3 Department of Colorectal Surgery, Kansai Medical University Medical Center, Moriguchi, Osaka, Japan; 4 Division of Vascular Surgery, Department of Surgery, Graduate School of Medicine, The University of Tokyo, Tokyo, Japan; 5 Division of Pathology, Cancer Institute, Japanese Foundation for Cancer Research, Tokyo, Japan

**Keywords:** advanced colorectal cancer, curative resection, vascular reconstruction

## Abstract

**INTRODUCTION:**

Although complete resection during radical surgery is a crucial prognostic factor for locally advanced colorectal cancer, achieving it is often difficult when the tumor invades the iliac artery system. Herein, we report a successful case requiring resection of the common iliac vessels and vascular reconstruction using a femoral–femoral arterial bypass (F–F bypass), with a comparison to 4 previous cases involving F–F bypass.

**CASE PRESENTATION:**

A 47-year-old male presented with advanced cecal cancer involving the right external iliac artery and vein, right femoral nerve, right ureter, right psoas muscle, and right iliacus muscle. He received systemic chemotherapy with a vascular endothelial growth factor inhibitor for 20 months, and percutaneous drainage of a psoas abscess was performed at a previous hospital. Following these interventions, he was referred to our hospital for radical resection. An F–F bypass was performed prior to abdominal surgery, and en bloc resection of the cecal cancer was subsequently achieved, encompassing the common iliac vessels, femoral nerve, iliacus muscle, psoas muscle, and ureter. The patient showed no signs of recurrence, graft infection, or occlusion 2 years postoperatively.

**CONCLUSIONS:**

This case demonstrates the potential of systemic chemotherapy followed by radical resection with extra-anatomical arterial bypass in achieving favorable long-term outcomes and satisfactory short-term results.

## Abbreviations

AFL
: aflibercept
BV
: bevacizumab
CIA
: common iliac artery
CIV
: common iliac vein
EIA
: external iliac artery
EIV
: external iliac vein
F–F
: femoral–femoral arterial bypass
FOLFIRI
: folinic acid/5-fluorouracil/irinotecan
FOLFOX
: folinic acid/5-fluorouracil/oxaliplatin
IIA
: internal iliac artery
IIV
: internal iliac vein
R0
: negative radial margin
RAM
: ramucirumab

## INTRODUCTION

Complete resection during radical surgery is a significant factor in achieving oncological control and improving patient prognosis in locally advanced colorectal cancer.^1)^ R0s have been strongly associated with reduced recurrence and improved survival rates in colorectal adenocarcinoma.^2)^ For patients with resectable colon cancers invading adjacent organs, complete en bloc multivisceral resection with sufficient margins is recommended for curative treatment.^3)^

However, achieving curative resection becomes particularly challenging in tumors involving the CIA or EIA. This is because the technical complexity of vascular reconstruction introduces potentially life-threatening complications, such as vascular graft infection or occlusion.^4,5)^ Careful patient selection is therefore crucial to ensure the feasibility of curative resection without recurrence.

However, determining the optimal indications and techniques remains unclear due to the rarity of cases requiring this procedure, thereby limiting the establishment of evidence-based guidelines. In this context, reporting rare but successful cases of vascular reconstruction in advanced tumors becomes valuable.

Herein, we report a successful case of colon cancer treated with vascular reconstruction using a F–F bypass. Additionally, we compare our patient with 4 other cases who underwent radical resection of primary or recurrent colorectal cancer.

## CASE PRESENTATION

### Patient information

**Fig. 1:**
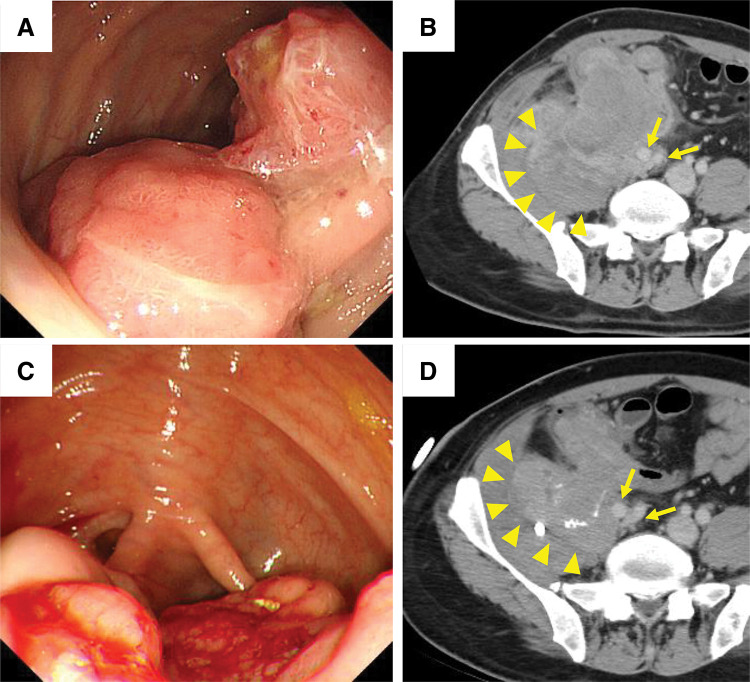
(**A**) Pre-chemotherapy colonoscopy shows a type 2 tumor in the cecum. (**B**) CT shows invasion of the external iliac vessels (arrows) and an abscess in the psoas muscle (arrowheads). (**C**) Post-chemotherapy colonoscopy shows shrinkage of the type 2 tumor. (**D**) CT shows shrinkage of the psoas muscle abscess (arrowheads); however, invasion of the external iliac vessels (arrows) remains.

A 47-year-old male presented with lower abdominal and right femoral pain. Colonoscopy revealed a type 2 tumor in the cecum, with a pathological diagnosis of well-differentiated tubular adenocarcinoma (**Fig. 1A**). CT showed that the tumor had invaded the right EIA and EIV, femoral nerve, ureter, and the psoas (with an accompanying psoas abscess) and iliacus muscles (**Fig. 1B**). Some swollen mesenteric lymph nodes were observed, suggesting lymph node metastases. The Union for International Cancer Control clinical stage was cT4bN1bM0 (corresponding to Stage IIIC), with no evidence of distant metastasis. Initially diagnosed as unresectable, the patient underwent 20 months of systemic chemotherapy. He initially underwent 35 courses of FOLFOX plus BV. Owing to an allergic reaction to oxaliplatin, the regimen was switched to FOLFIRI plus RAM, and he underwent this regimen for 4 courses. Subsequently, because of an allergic reaction to RAM, the regimen was changed again to FOLFIRI plus AFL, and he underwent the regimen for 3 courses. Percutaneous drainage of the psoas abscess was performed concurrently with chemotherapy. As a result of these interventions, the tumor size was reduced from 14 to 6.5 cm, and the infection with abscess formation was gradually controlled. These findings made conversion surgery possible, encouraging the patient to seek surgical treatment at our hospital.

**Fig. 2:**
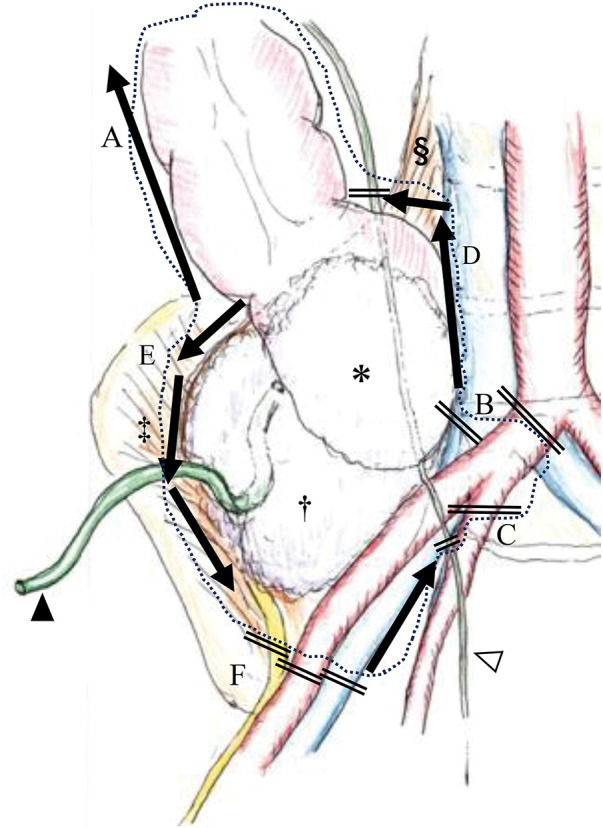
Schema of the tumor (*) invasion and abscess (**†**) with drainage tube (black arrowhead). The abscess extended to the psoas major (§) and iliacus muscles (**‡**). Black arrows indicate the planned resection lines, and the dotted line indicates the actual extent of resection. Vessel ligation sites are marked with black double lines. (**A**) The hepatic flexure of the colon was mobilized laparoscopically. (**B**) The right common iliac artery and vein were ligated proximally. (**C**) The right internal iliac artery and vein were ligated. (**D**) The major psoas muscles were dissected from the vertebral body, and (**E**) the iliacus muscles were dissected from the ilium. (**F**) The distal external iliac artery, vein, and femoral nerve were ligated, and the ureter (white arrowhead) was resected.

On initial evaluation, he reported no abdominal pain, and a small amount of pus was observed from the drainage tube. He had no comorbidities and was classified as having American Society of Anesthesiologists Physical Status II. Laboratory testing revealed mild inflammation (white blood cell count: 4080/µL, C-reactive protein: 4.46 mg/dL) and anemia requiring blood transfusion (hemoglobin: 7.6 g/dL). It revealed normal organ function. Colonoscopy and CT scan post-chemotherapy showed tumor shrinkage and reduction of the psoas abscess; however, persistent invasion of the right EIA, EIV, femoral nerve, ureter, and psoas and iliacus muscles remained (**Figs. 1C**, **1D**, and **2**). Based on these findings, we deemed the tumor resectable with radical resection, specifically recommending en bloc resection of the right CIA, CIV, femoral nerve, ureter, and psoas and iliacus muscles (**Fig. 2**).

### Surgical findings

**Fig. 3:**
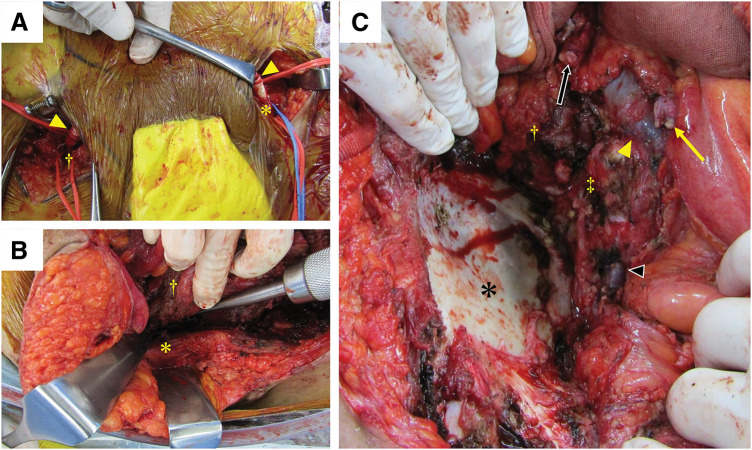
Operative findings. (**A**) Femoral–femoral crossover bypass between the left femoral (*) and right superficial femoral arteries (†). A 6-mm ring-reinforced prosthetic vascular graft was used (arrowheads). (**B**) Resection of the iliacus muscle (†) from the surface of the ilium (*). (**C**) View following tumor resection, showing the surface of the ilium (*), vertebral body (‡), and the transected end of the psoas major muscle (†). The stumps of the common iliac artery (yellow arrow), common iliac vein (yellow arrowhead), internal iliac vein (black arrowhead), and ureter (black arrow) are also visible.

A multidisciplinary surgical team comprising colorectal, urological, vascular, and orthopedic surgeons performed the procedure (**Fig. 2**). First, the colorectal surgeons performed staging laparoscopy, confirming the absence of peritoneal dissemination or liver metastasis. Using a 6-mm ring-reinforced prosthetic vascular graft made of expanded polytetrafluoroethylene, the vascular surgeon subsequently performed an F–F bypass from the left femoral artery to the right superficial femoral artery (**Fig. 3A**). The bilateral inguinal incisions were carefully separated from the laparotomy site during the bypass procedure, then they were cleanly closed and dressed with waterproof dressing material immediately after vascular reconstruction to minimize the risk of contamination. Laparoscopically, the hepatic flexure of the colon was mobilized, and central ligation of the ileocolic vessels was performed. Laparotomy was then performed for the remaining resection. The right CIA and CIV were ligated proximally, followed by ligation of the right IIA and IIV. In collaboration with the orthopedic surgeon, the major psoas and iliacus muscles were dissected from the vertebral body and ilium, respectively (**Fig. 3B**). Following muscle resection, the distal EIA, EIV, and femoral nerve were ligated, and the ureter was resected. In addition, the abdominal wall containing the abscess and drainage tube was included in the scope of the resection (**Fig. 3C**). Lastly, ileum transverse colon anastomosis (functional end-to-end anastomosis) was performed by the colorectal surgeons and ileal ureter interposition for urinary tract reconstruction was performed by the urological surgeons. The estimated blood loss was 4215 mL, and the operative time was 763 min.

### Pathological findings

**Fig. 4:**
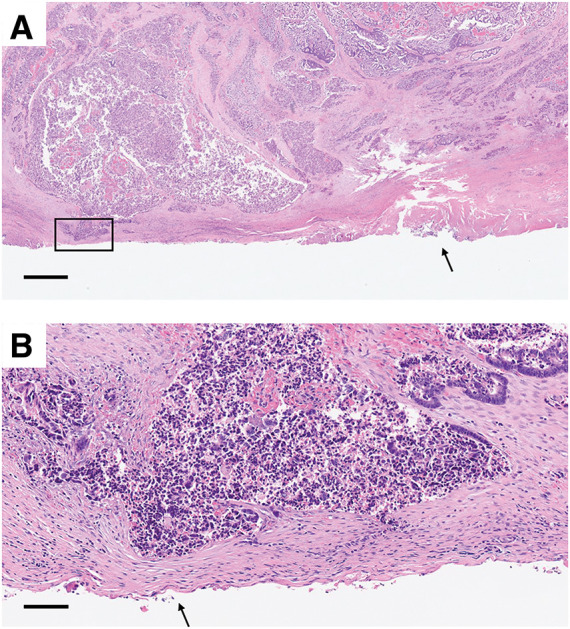
Histopathological images of the resection margin. (**A**) Low magnification. Crushing artifacts are observed near the dissection plane (black arrow). The area enclosed by the black square corresponds to the region close to the resection margin. The black line represents a scale bar of 1 mm. (**B**) High magnification view of the area outlined by the black square in (**A**). The black arrow indicates the site where the tumor was closest to the resection margin, measuring 0.08 mm. The black line represents a scale bar of 0.1 mm.

Histopathological examination revealed a moderately to well-differentiated tubular adenocarcinoma with invasion into the psoas and iliacus muscles. There was no invasion into the iliac vessels. While the resection margin at the iliacus muscle was narrow (**Table 1**), it was ultimately negative for tumor involvement (**Fig. 4**). Lymph node metastasis was not identified.

**Table 1:** Cases that required curative resection and vascular reconstruction at our institution

	Age	Sex	Diagnosis	Preoperative treatment	Operative procedure	Arterial reconstruction method	Operative time	Blood loss	Resected vessels	Resected organs	Pathological findings	Margin
Case 1	47	Male	Primary cecal cancer	FOLFOX + BV FOLFIRI + RAM FOLFIRI + AFL 20 months	Ileocecal resection	F–F bypass	763 min	4215 mL	Right CIA/CIV	Ureter Psoas major muscle Iliacus muscle Right femoral nerve	pT4b (psoas major muscle, iliacus muscle), N0	Negative (0.08 mm, iliacus muscle)
Case 2	63	Female	Primary sigmoid colon cancer	None	Sigmoidectomy	F–F bypass	684 min	5050 mL	Left EIA	Ureter Ileum and cecum Psoas major muscle Uterus Bilateral ovaries	pT4b (rectum, ascending colon, ileum), N0	Negative (inflammatory reaction)
Case 3	52	Female	Primary appendiceal cancer	CAPOX 7 months	Ileocecal resection	F–F bypass	531 min	990 mL	Right EIA/EIV	Psoas major muscle Iliacus muscle Right femoral nerve Ilium (partial)	pT4b (psoas major muscle, iliacus muscle, skin), N2a	Negative (8 mm, iliacus muscle)
Case 4	55	Male	Primary appendiceal cancer	CAPOX 1.5 months	Ileocecal resection	F–F bypass	777 min	2650 mL	Right EIA/EIV	Ureter Bladder (partial) Psoas major muscle	pT4b (bladder, retroperitoneum, ileum), N1b	Negative (0.5 mm, psoas muscle)
Case 5	71	Female	Recurrent rectal cancer (right LPLN metastasis)	FOLFOX + BV 1 month	Resection of LPLN	F–F bypass	922 min	110 mL	Right EIA/EIV	Psoas major muscle Internal obturator muscle Obturator nerve	Recurrent rectal cancer with invasion of the psoas major and internal obturator muscles	Unassessable in internal obturator muscle

AFL, aflibercept; BV, bevacizumab; CAPOX, capecitabine/oxaliplatin; CIA, common iliac artery; CIV, common iliac vein; EIA, external iliac artery; EIV, external iliac vein; F–F bypass, femoral-femoral arterial bypass; FOLFIRI, folinic acid/5-fluorouracil/irinotecan; FOLFOX, folinic acid/5-fluorouracil/oxaliplatin; LPLN, lateral pelvic lymph node; RAM, ramucirumab

### Postoperative course

Oral intake was initiated on postoperative day 6. On postoperative day 15, the patient developed a fever, and a CT scan showed a new abdominal abscess on the iliac surface. Percutaneous drainage and intravenous antibiotics successfully managed the abscess.

He received physical therapy for motor weakness of the right lower limb due to resection of the femoral nerve, major psoas, and iliacus muscles. He regained ambulation with the aid of a single-handed cane. His right lower limb edema, which initially appeared due to EIV resection without venous reconstruction, improved over time. The patient was discharged on postoperative day 57 without antibiotics and transitioned back to independent living. There has been no evidence of recurrence 2 years postoperatively.

## DISCUSSION

This report describes the successful curative resection by creating an extra-anatomical F–F bypass for locally advanced cecal cancer invading the CIA, consequently achieving favorable clinical and oncological outcomes.

Our experience includes a total of 5 cases, including the present case, that required vascular reconstruction due to resection of the CIA or EIA (**Table 1**). While all cases achieved favorable short-term outcomes and curative resection (**Tables 1** and **2**), Cases 3 and 4 experienced early tumor recurrence. Moreover, Case 5 developed pneumonia shortly after discharge and passed away, despite no cancer recurrence (**Table 2**). Case 5, a 71-year-old woman, experienced edema in the right lower limb owing to surgery after discharge, which led to a decline in her general condition, malnutrition, and ultimately fatal pneumonia. Considering the indications for locally advanced cancers, as in our series, careful decision-making with thorough discussion from various aspects in multidisciplinary team conferences should be made. In Case 2, pathological findings of the resected specimens revealed malakoplakia, an inflammatory change, in the area that was initially suspected to be tumor invasion on preoperative imaging. This is consistent with the difficulties encountered in the preoperative diagnosis of tumor invasion. In fact, several studies have reported a significant mismatch (40%–50%) between clinical and pathological T4b categorization, suggesting potential overtreatment in suspected cases.^6)^

**Table 2:** Short- and long-term outcomes in cases that required curative resection and vascular reconstruction at our institution

	Postoperative complications	Postoperative hospital stay	Recurrence	DFS or RFS	Prognosis
Case 1	Seroma	57 days	None	26 months (DFS)	Alive
Case 2	Ileus	21 days	None	15 months (DFS)	Alive
Case 3	SSI	25 days	Peritoneum	5 months (RFS)	Alive (23 months)
Case 4	Chylous ascites	33 days	Liver, lung	2 months (RFS)	Dead (20 months)
Case 5	Seroma	44 days	None	5 months (DFS)	Dead (5 months)

DFS, disease-free survival; RFS, recurrence-free survival; SSI, surgical site infection

Regardless of pathological organ involvement, preoperative systemic chemotherapy with molecular targeting agents may be necessary to achieve tumor shrinkage and improve oncological outcomes. In Case 1, the patient underwent a 20-month regimen of FOLFOX + BV, FOLFIRI + RAM, and FOLFIRI + AFL. Similar approaches are employed for initially unresectable cases with synchronous and metachronous liver metastases, where radical resection often follows systemic chemotherapy.^7)^ The effectiveness of preoperative chemotherapy for locally advanced colon cancer has been reported in terms of achieving complete resection and improved disease control within 2 years.^8)^ Moreover, a retrospective analysis of the National Cancer Database exhibited improved survival among patients with cT4b colon cancer treated with neoadjuvant chemotherapy compared to those treated with adjuvant chemotherapy.^9)^ The potential for underlying systemic disease at the time of initial diagnosis in these locally advanced cases emphasizes the need for chemotherapy and invasive surgery. However, the significant surgical burden may limit the feasibility of adjuvant chemotherapy due to prolonged recovery periods.^10)^ Thus, preoperative systemic chemotherapy followed by radical resection may be the optimal strategy for controlling distant metastasis, especially in locally advanced tumors. In these cases, postoperative adjuvant chemotherapy was not administered owing to the prolonged postoperative recovery. In our department, for locally advanced colon cancer, neoadjuvant chemotherapy is considered when organ preservation is expected. One report recommended a neoadjuvant chemotherapy period 6 weeks, followed by 18 weeks of adjuvant chemotherapy for locally advanced colon cancer.^8)^ However, in cases where postoperative adjuvant chemotherapy containing oxaliplatin (known for its high risk of neuropathy) may not be well tolerated due to edema following venous resection or gait impairment from muscle and nerve resection, 6 months of preoperative chemotherapy should be considered.

Preoperative chemoradiotherapy was not selected because, unlike rectal cancer, adequate surgical fields can typically be secured in colon cancer, allowing for negative resection margins through combined resection. Additionally, there is a risk of radiation exposure to the small intestine. Although not available at our institution, intraoperative radiotherapy may be a viable option in facilities where it is available, particularly in cases where securing the margin is difficult.^11)^

The present case highlights the significance of a multidisciplinary surgical approach. In Case 1, negative resection margins were achieved at the iliacus muscle due to the experience of the orthopedic surgeon. If the colorectal surgeons had operated in this area, their lack of experience might have resulted in positive resection margins. This emphasizes the value of including relevant specialists and ensuring proper lateral landmarking, such as the iliacus in this case, when managing complex resections involving adjacent organs or tissues.

In vascular reconstruction, graft infection can lead to a life-threatening situation. Fortunately, none of the 5 patients in our series, including the present case, experienced this complication. In all cases, vascular reconstruction and intra-abdominal colorectal surgery were performed simultaneously but separately. Additionally, prosthetic vascular grafts were used in all cases. Autologous grafts, including saphenous vein grafts, are generally preferred in potentially contaminated fields, such as colorectal surgery, due to a lower risk of graft infection and failure.^4,5)^ However, the use of prosthetic vascular grafts was possible in our case by limiting the vascular reconstruction surgical site to the inguinal area, thereby minimizing the risk of infection from the colorectal surgical site. We also performed vascular reconstruction before resection of the bowel and urinary tract to prevent infection of the prosthetic grafts. Previous reports have described simultaneous vascular reconstruction similar to our method,^12–15)^ and one study recommended separating the dates for vascular reconstruction and tumor resection.^16)^ Regarding the vascular reconstruction route, extra-anatomical F–F or axillofemoral bypasses are preferred as they avoid the abdominal cavity, whereas some reports utilize intra-abdominal *in situ* reconstruction for the aorta^11,14)^ or employ autologous grafts *in situ*.^4)^ When the surgical field is contaminated due to bowel resection, the extra-anatomical route is beneficial in preventing graft infection.^12)^ Additionally, since we did not require aortic reconstruction in our case, we selected F–F bypass for the same-day surgery using a prosthetic vascular graft. Regarding antibiotic management, we administered prophylactic cefmetazole for approximately 1 week in patients without pre-existing infections. For patients with pre-existing abscesses or similar infections, we initiated antibiotic treatment based on microbiological culture results prior to surgery and continued treatment for approximately 6 weeks.

Interestingly, venous reconstruction was not performed in Case 1 despite CIV resection. This is consistent with previous studies suggesting that collateral flow can develop following EIV resection, potentially eliminating the need for venous reconstruction.^12,13)^ Indeed, postoperative contrast-enhanced CT in Case 1 confirmed adequate blood flow return through collateral pathways between the right and left IIVs. Furthermore, a soft tissue sarcoma study comparing patients undergoing arterial reconstruction alone with those undergoing bilateral arteriovenous reconstruction for limb salvage surgery did not identify a clear benefit for routine venous reconstruction after vascular resection.^17)^

## CONCLUSIONS

Our study demonstrates the feasibility of achieving favorable short-term and long-term outcomes in carefully selected patients with advanced locally invasive colon cancer through a multidisciplinary approach. Combined CIA and EIA resection with extra-anatomical arterial reconstruction using an F–F bypass represents a viable surgical strategy. For cases requiring multi-organ resection, meticulous preoperative evaluation is essential to determine the indications for radical resection and to guide optimal surgical planning.

## ACKNOWLEDGMENTS

None.

## DECLARATIONS

### Funding

None.

### Authors’ contributions

TN: Investigation, writing of the original draft, review, and editing.

YF, TT, TS, SM, TM, TY, MT, TA: Supervision, writing, review, and editing.

All authors have read and approved the manuscript.

### Availability of data and materials

Not applicable.

### Ethics approval and consent to participate

This work does not require ethical considerations or approval. Written informed consent was obtained from the patient.

### Consent for publication

Informed consent for publication of this case report was obtained from the patient.

### Competing interests

The authors declare no conflicts of interest.
